# The Tumor–Platelet–Immune Interface: Driving Metastasis, Pre-Metastatic Niche Formation and Therapeutic Vulnerabilities

**DOI:** 10.1017/erm.2026.10043

**Published:** 2026-04-07

**Authors:** Jalal Naghinezhad, Ehsan Kamali Yazdi, Ahmad Mohajerian, Negin Yousefi Chermehini, Pedram Ghanavati, Hamid Motamedi, Hadi Rezaeeyan

**Affiliations:** 1 Department of Medical Laboratory Sciences, School of Allied Medical Science, Mazandaran University of Medical Sciences, Sari, Iran; 2 Alborz University of Medical Sciences, Alborz, Iran; 3 Department of Emergency Medicine, Faculty of Medicine, Ahvaz Jundishapur University of Medical Sciences, Ahvaz, Iran; 4 Department of Neurosurgery, Firouzgar Hospital, Iran University of Medical Sciences (IUMS), Tehran, Iran; 5 Asadabad School of Medical Sciences, Asadabad, Iran

**Keywords:** circulating tumor cells, enzyme- and pH-sensitive nanoparticle, hydrogel, neutrophil extracellular traps, platelet-leukocyte crosstalk, pre-metastatic niche, tumor microenvironment

## Abstract

Metastatic dissemination remains the leading cause of cancer-related mortality, driven not only by tumor-intrinsic factors but also by dynamic interactions within the tumor microenvironment (TME). Platelets and leukocytes orchestrate a systemic pro-metastatic network by shielding circulating tumor cells (CTCs), inducing neutrophil extracellular trap (NET) formation, and remodeling the extracellular matrix to prime pre-metastatic niches. This platelet-leukocyte crosstalk simultaneously promotes immune evasion, thromboinflammation, and metastatic seeding, creating a multi-cellular, temporally coordinated program that conventional anti-platelet and anti-inflammatory therapies inadequately target. Here, we propose a next-generation, multi-stimuli-responsive nanoparticle platform designed to disrupt these interconnected metastatic circuits. Engineered to respond to acidic pH in primary tumors and matrix metalloproteinases in pre-metastatic niches, these nanoparticles enable spatiotemporally controlled release of cytotoxic agents, anti-platelet drugs, and immune checkpoint inhibitors. Surface functionalization with anti-P-selectin further enhances specificity to tumor vasculature and facilitates platelet ‘hitchhiking’ for targeting CTCs. By simultaneously neutralizing platelet-leukocyte interactions, inhibiting NET-mediated scaffolds, and restoring anti-tumor immunity, this integrated strategy addresses multiple pro-metastatic mechanisms in a coordinated fashion. This work provides a conceptual and translational framework for precision anti-metastatic therapeutics, transforming the paradigm from single-pathway interventions to network-targeted strategies that disrupt tumor progression, CTC survival and metastatic niche formation. Our approach represents a critical step toward actionable, multi-modal interventions capable of preventing metastatic disease.

## Graphical abstract

**Figure:**
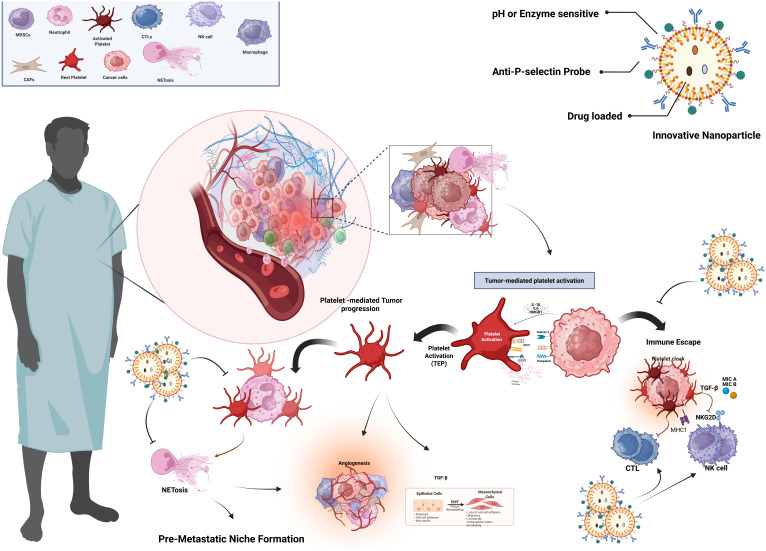

## Highlights

1. Platelet-leukocyte crosstalk promotes CTC survival, immune evasion and pre-metastatic niche priming.
2. NETosis facilitates metastatic progression by trapping CTCs and remodeling the extracellular matrix.
3. Conventional anti-platelet and anti-inflammatory therapies incompletely target these interconnected pathways.
4. Multi-stimuli-responsive nanoparticles and functionalized hydrogels allow spatiotemporally precise delivery of cytotoxic, anti-platelet and immunomodulatory agents.
5. Multi-modal network disruption integrates platelet, immune and tumor targeting to limit metastatic seeding and disease progression.

## Introduction

### Tumor microenvironment (TME) as a driver of cancer progression

Cancer progression and metastasis are increasingly recognized not solely as properties of malignant cells, but as emergent phenomena orchestrated by the TME (Refs 1, 2, 3). The TME comprises a complex and dynamic network of stromal cells, immune populations, extracellular matrix components, and soluble mediators that collectively shape tumor growth, immune escape, and metastatic dissemination (Refs 1, 4). Tumor cells exploit this niche to modulate angiogenesis, suppress anti-tumor immunity, and prime distant organs for metastatic colonization, creating pre-metastatic niches that are permissive for circulating tumor cell (CTC) engraftment (Refs 5, 6, 7). This perspective shifts the focus from tumor-centric therapies to microenvironment-oriented interventions, emphasizing the need to decode the cellular and molecular circuits that underpin metastatic progression.

### Platelets and leukocytes: Beyond hemostasis and immunity

Platelets and leukocytes, traditionally studied in the contexts of hemostasis and innate immunity, are now recognized as active collaborators in tumor progression (Refs 8, 9). Platelets form protective cloaks around CTCs, shielding them from immune clearance and facilitating adhesion to endothelium (Refs 9, 10). Leukocytes, particularly neutrophils, engage in NETosis, releasing extracellular traps that both physically capture CTCs and remodel the extracellular matrix to promote metastatic seeding (Refs 11, 12). The bidirectional crosstalk between platelets and leukocytes amplifies immunosuppressive signaling, modulates the phenotype of macrophages and T-cells, and creates a systemic thromboinflammatory network (Ref. 13). Together, these interactions constitute a multi-level metastatic engine, driving tumor survival, dissemination, and pre-metastatic niche formation.

### The clinical burden of metastasis and the unmet need for targeted TME therapies

Metastasis remains the principal cause of cancer-related mortality, yet current therapeutic paradigms are largely tumor cell–centric, failing to address the systemic, TME-driven processes that enable CTC survival and organ colonization. Conventional anti-platelet or anti-inflammatory approaches offer partial mitigation but are limited by systemic toxicity, lack of spatiotemporal precision, and failure to disrupt the integrated platelet-leukocyte metastatic network. This critical gap underscores an urgent need for precision-targeted, multi-modal interventions that modulate the TME, intercept CTCs, and prevent pre-metastatic niche formation. Here, we propose innovative strategies leveraging stimuli-responsive nanoparticles, functionalized to exploit tumor- and niche-specific cues, capable of delivering cytotoxic, anti-platelet and immunomodulatory payloads in a coordinated, network-targeted manner. By integrating mechanistic insights with advanced biomaterials, such approaches promise to transform our ability to prevent metastasis and redefine therapeutic paradigms in oncology.

## Platelet-leukocyte crosstalk in the TME

Within the TME, platelets and leukocytes form a highly coordinated, pro-tumorigenic network that transcends their classical roles in hemostasis and innate immunity (Ref. 14). This axis orchestrates a complex interplay of immune evasion, metastatic dissemination and systemic immunomodulation, effectively converting normally protective immune mechanisms into facilitators of tumor progression (Ref. 15). Tumors exploit platelet-leukocyte crosstalk to create a dynamic protective niche that shields malignant cells from immunosurveillance, enhances vascular adhesion, primes pre-metastatic sites and facilitates distant organ colonization (Ref. 16). The result is a multi-layered, self-reinforcing network that integrates inflammation, coagulation and immune suppression to favor tumor survival and dissemination.

### Mechanisms of platelet-leukocyte interactions

The molecular and cellular dialogue between platelets and leukocytes is both structurally intimate and functionally sophisticated, combining direct cell–cell interactions with a rich milieu of soluble mediators (Ref. 17). Platelets express adhesion molecules – P-selectin, CD40L, and integrins (αIIbβ3) – that engage PSGL-1, CD40, and integrins on neutrophils, monocytes, and lymphocytes (Ref. 18). These interactions initiate bidirectional activation, whereby leukocytes acquire immunosuppressive and pro-thrombotic phenotypes, while platelets are further primed to release cytokines and growth factors.

A central consequence of this crosstalk is neutrophil extracellular trap (NET) formation, which not only traps tumor cells but also amplifies local oxidative stress and inflammation (Ref. 19). Simultaneously, monocytes and macrophages polarize toward tumor-supportive, immunosuppressive states, while T-cells become functionally exhausted through inhibitory signals (Refs 20, 21).

Soluble platelet-derived mediators – CXCL4, CCL5 and thromboxane A2 – act synergistically to enhance leukocyte recruitment, modulate immune phenotype and facilitate adhesion to tumor cells and endothelial surfaces (Ref. 22). This combination of adhesive, paracrine, and redox-mediated signaling establishes a spatiotemporally coordinated microenvironment in which tumor cells are shielded, vascular interactions are stabilized, and metastatic dissemination is facilitated (Refs 23, 24).

The TME, therefore, becomes a self-perpetuating ecosystem: platelet-leukocyte aggregates reinforce immunosuppression, promote thrombosis, prime pre-metastatic niches and create physical and biochemical barriers to immune cell infiltration (Ref. 4). By hijacking these physiological processes, tumors gain a multidimensional survival advantage, ensuring both local persistence and systemic dissemination.

### Contribution to tumor immune evasion

Platelet-leukocyte crosstalk constitutes a highly adaptive immune evasion mechanism exploited by tumors to survive both within the primary microenvironment and during systemic dissemination (Ref. 25). Platelet–leukocyte aggregates act as dynamic protectors of CTCs, masking tumor-associated antigens and preventing immune recognition by NK cells, cytotoxic T lymphocytes and other innate effectors (Ref. 26). This mechanical protection is reinforced by biochemical signaling: platelets release TGF-β (Ref. 27), platelet factor 4 (CXCL4) (Ref. 28), and ATP, while associated leukocytes upregulate PD-L1, IL-10, and other immunosuppressive mediators, collectively inducing T-cell exhaustion, NK cell inhibition and suppression of antigen-presenting cell activity (Ref. 29).

Simultaneously, platelet-derived signals reprogram innate immune cells in circulation and at distant sites (Ref. 18). Monocytes and macrophages adopt a tumor-supportive M2 phenotype, secreting angiogenic and extracellular matrix remodeling factors (Refs 30, 31). This creates a pre-metastatic niche, rich in immunosuppressive and pro-thrombotic cues, which lowers the barrier for tumor extravasation, colonization, and secondary outgrowth.

Platelet–leukocyte crosstalk functions as a multi-dimensional mediator of tumor progression, integrating local immune evasion with systemic niche priming (Refs 32, 33). Platelet–leukocyte crosstalk drives tumor progression by stabilizing CTCs, masking tumor antigens from NK and T cells, suppressing immune effectors and polarizing macrophages toward pro-tumor phenotypes. Systemically, these interactions prime pre-metastatic niches, facilitating metastatic colonization. Table 1 summarizes these mechanistic pathways and distinguishes established processes from proposed therapeutic interventions, highlighting actionable vulnerabilities in CTC survival and metastasis.

**Table 1.** Mechanistic roles of platelet–leukocyte crosstalk in tumor progression and therapeutic opportunities

Axis of crosstalk	Key molecular mediators	Mechanistic consequences	Tumor advantage/immune evasion	Therapeutic intervention (proposed/established)
Adhesive interactions (platelet–leukocyte aggregates)	P-selectin–PSGL–1, CD40L–CD40, αIIbβ3–Mac–1	Stabilizes heterotypic aggregates; bidirectional activation	Sustains thromboinflammatory microenvironments; enhances CTC survival	**Established:** integrin or P-selectin blockade; **Proposed:** anti-CD40L
Paracrine immunomodulation	TGF-β, CXCL4, CCL5, thromboxane A2, ATP	Suppresses cytotoxic T cell and NK activity; recruits immunosuppressive leukocytes	Effector cell exhaustion; tumor-supportive immune skewing	**Established:** TGF-β inhibition; **Proposed:** chemokine receptor antagonists, purinergic blockade
NETosis induction	Platelet-derived HMGB1, P-selectin, thrombin, histones	Triggers neutrophil NET release; ECM remodeling; vascular adhesion scaffolds	Captures CTCs; promotes extravasation; protects from immune clearance	**Proposed:** PAD4 inhibitors, DNases, anti-HMGB1 therapy
Monocyte/macrophage polarization	CD40L, CXCL4, TGF-β, platelet microparticles	Shifts toward M2-like, pro-angiogenic macrophages	ECM remodeling, angiogenesis, pre-metastatic niche priming	**Proposed:** CSF–1R inhibition, microparticle neutralization
Adaptive immune suppression	PD-L1 upregulation on leukocytes, TGF-β, IL–10	Inhibits cytotoxic T cells; impairs antigen presentation	Tumor immune evasion	**Established:** checkpoint inhibitors (anti-PD–1/PD-L1, anti-CTLA–4)
Platelet coating of CTCs (‘platelet shield’)	GPIIb/IIIa–fibrinogen, P-selectin, integrins, CD40L	Masks tumor-associated antigens; recruits leukocytes	Protects CTCs from NK and T cell clearance	**Established:** P2Y12, GPIIb/IIIa inhibitors; **Proposed:** P-selectin antagonists
Pre-metastatic niche priming	MMPs, VEGF, TGF-β, platelet microparticles, NETs	ECM remodeling, immunosuppression, angiogenesis at distant sites	Lowers threshold for metastatic colonization	**Proposed:** MMP inhibitors, anti-angiogenics, targeting platelet-derived extracellular vesicles

### Platelet clock: Circulating tumor cells and immune shielding

Upon activation, platelets upregulate adhesion molecules such as P-selectin, enabling them to sequentially bind to CTCs and other immune cells. This temporally coordinated interaction, referred to as the ‘platelet–CTC clock’, facilitates CTC survival, immune evasion, and metastatic potential (Refs 34, 35). Upon intravasation, tumor cells are immediately coated by platelets, forming transient platelet cloaks that serve both mechanical and biochemical protective functions (Ref. 36). These cloaks mask tumor-associated antigens, preventing recognition by NK cells, cytotoxic T lymphocytes, and complement-mediated lysis, while simultaneously delivering platelet-derived immunosuppressive signals such as TGF-β, ATP, and CD40L, which modulate leukocyte activity in real time (Ref. 37).

Platelets also actively coordinate leukocyte hitchhiking, recruiting neutrophils and monocytes to CTCs (Refs 38, 39). Neutrophils undergo NETosis, releasing NETs that physically entangle tumor cells, promote adhesion to endothelial surfaces and enhance extravasation into pre-metastatic niches (Ref. 40). Monocytes and macrophages are polarized toward tumor-supportive phenotypes, secreting matrix metalloproteinases and growth factors that prepare distant tissues for colonization (Ref. 41). This temporally orchestrated interaction – the platelet clock – extends the survival window of CTCs, ensures immune shielding during circulation and establishes a mobile, systemic metastatic network capable of initiating pre-metastatic niche formation.

**Figure 1.**
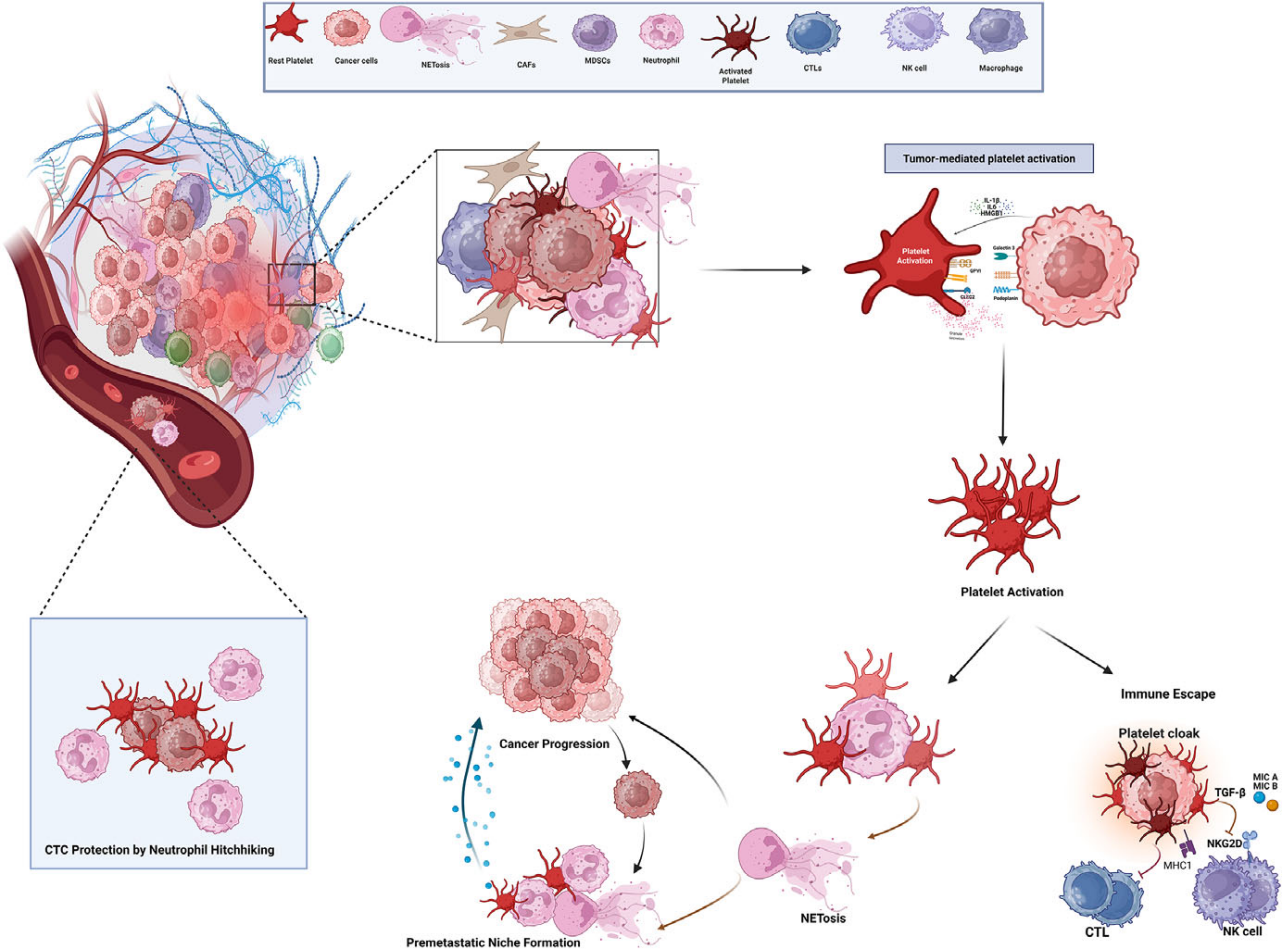
Mechanistic integration of platelet–leukocyte–tumor cell crosstalk in tumor progression and metastasis. *Note*: Platelets are activated within the tumor microenvironment, forming heterotypic aggregates with both neutrophils and circulating tumor cells (CTCs). Established mechanisms include platelet–tumor aggregates masking tumor antigens to evade NK and cytotoxic T cell recognition, and platelet–neutrophil interactions triggering NETosis, which promotes ECM remodeling, vascular adhesion and pre-metastatic niche formation. In circulation, CTCs are further protected through neutrophil hitchhiking and platelet coating, enhancing survival under shear stress and immune attack. Figure 1 synthesizes these interactions into a unified mechanistic framework, highlighting points where therapeutic intervention could disrupt CTC survival and metastatic dissemination.

Critically, the platelet clock is not merely a passive process; it represents a programmable, targetable system in which the temporal and spatial coordination of platelets, leukocytes and CTCs defines the metastatic trajectory (Ref. 42). Disrupting this clock, by interfering with platelet-leukocyte engagement, NET formation or platelet-derived immunosuppressive signaling, emerges as a high-priority therapeutic strategy to prevent metastasis and dismantle the protective circulatory niche for tumor cells (see Figure 1).

## NETosis and neutrophil-mediated metastatic facilitation

Neutrophils have emerged as influential regulators of tumor progression and metastatic dissemination, yet their role is neither uniform nor universally pro-metastatic across cancer types (Ref. 43). Within both the TME and systemic circulation, neutrophils can undergo NETosis, releasing chromatin fibers decorated with histones, proteases and inflammatory mediators (Ref. 44). While numerous studies associate NET formation with enhanced immune evasion, vascular adhesion and metastatic seeding (Ref. 32), an important unresolved question remains whether NETosis is a causal driver of metastasis or a secondary byproduct of advanced tumor-associated inflammation. Indeed, the majority of evidence supporting a pro-metastatic role for NETs is derived from preclinical models and correlative clinical observations, underscoring the need for cautious interpretation.

Importantly, NETosis does not exert uniform effects across malignancies. While robust pro-metastatic roles have been demonstrated in breast, pancreatic, and colorectal cancers (Refs 32, 43), other tumor types exhibit neutral or even context-dependent responses to neutrophil activation. In certain immune-infiltrated tumors, NET-associated proteases may paradoxically enhance antigen release and immune recognition, suggesting that NETs can exert divergent biological effects depending on tumor immunogenicity, stromal composition and vascular architecture (Refs 13, 45). These discrepancies highlight a major conceptual gap in the field: NETosis is often discussed as a binary pro-tumor mechanism, despite substantial evidence that its functional consequences are spatially, temporally, and disease-stage dependent.

### NET formation in the TME and pre-metastatic niches: Drivers, variability and unresolved mechanisms

Tumor-derived mediators such as granulocyte colony-stimulating factor (G-CSF), IL-8, high-mobility group box 1 (HMGB1), and platelet-released chemokines (CXCL4, CCL5) have been shown to prime neutrophils toward NETosis both locally and at distant pre-metastatic sites (Ref. 46). However, the responsiveness of neutrophils to these signals is highly heterogeneous, shaped by host inflammatory status, metabolic stress, oxidative signaling thresholds, and prior immune conditioning (Refs 47, 48, 49). This heterogeneity may explain why NET formation is robust in some metastatic settings but absent or transient in others.

NET-mediated remodeling of the extracellular matrix (ECM) has been proposed to expose integrins, selectins, and adhesion molecules that facilitate CTC arrest (Ref. 50). While compelling, much of this evidence relies on static imaging and endpoint analyses, leaving unresolved whether NETs actively initiate pre-metastatic niche formation or merely stabilize niches already conditioned by tumor-derived extracellular vesicles and cytokines. Furthermore, although NET-associated proteins recruit monocytes, macrophages, platelets and endothelial progenitors (Ref. 51), it remains unclear whether these recruited cells require NET scaffolds for functional integration or whether NETs simply amplify pre-existing inflammatory gradients.

Platelet–neutrophil crosstalk further complicates causal inference. Platelet P-selectin and CD40L interactions with neutrophil PSGL-1 and CD40 clearly enhance NETosis through ROS generation and chromatin decondensation (Refs 52, 53). Yet, platelet activation itself is strongly influenced by tumor burden and systemic inflammation, raising the possibility that NET-rich environments reflect advanced disease states rather than early metastatic triggers. This bidirectional amplification loop, while mechanistically elegant, blurs the distinction between cause and consequence in metastatic progression (Ref. 54).

### NET-mediated tumor cell entrapment and thrombosis: Association versus dependency

NETs are frequently described as physical scaffolds that entrap CTCs within the circulation, increasing endothelial contact time and facilitating adhesion under shear stress (Refs 12, 38, 55). Although experimental disruption of NETs reduces CTC adhesion in several models (Refs 56, 57), it remains uncertain whether NETs are strictly required for metastatic arrest or whether they function as one of multiple redundant adhesive systems. Selectins, integrins and platelet-derived fibrin networks can independently mediate similar effects, raising questions about the relative contribution of NETs in vivo.

NET-associated histones and proteases potently activate platelets and tissue factor pathways, promoting microthrombi formation around trapped tumor cells (Ref. 58). This thromboinflammatory niche has been implicated in immune evasion by shielding CTCs from NK cell and cytotoxic T-cell clearance (Ref. 59). However, clinical attempts to target thrombosis or platelet activation have yielded mixed outcomes, with antiplatelet therapies showing limited efficacy and unacceptable bleeding risks in oncologic settings. These translational failures suggest that NET-driven thrombosis, while mechanistically important, may not be easily disentangled from essential hemostatic processes.

Thus, the tumor–platelet–neutrophil axis represents a highly adaptive but therapeutically challenging metastatic hub. While NET disruption may reduce microthrombus stability and immune shielding, the redundancy of pro-thrombotic pathways and compensatory inflammatory circuits remains a major obstacle to clinical translation.

### Neutrophil hitchhiking: Enhancing CTC survival and extravasation

The concept of ‘neutrophil hitchhiking’, wherein CTCs physically associate with neutrophils to enhance survival and extravasation, has gained increasing attention (Ref. 38). These heterotypic clusters confer resistance to shear stress, anoikis and immune surveillance (Refs 35, 60). Nevertheless, most supporting data are derived from snapshot analyses of circulating clusters, leaving open the possibility that these interactions preferentially occur among the most aggressive tumor clones rather than actively generating metastatic potential.

Neutrophils within these clusters release ROS, proteases and cytokines that may create a localized immunosuppressive microenvironment (Ref. 44). However, whether these signals actively reprogram CTCs or simply reflect neutrophil activation remains unresolved. Moreover, neutrophil-dependent enhancement of platelet deposition and microthrombi formation (Ref. 54) raises a key unanswered question: are neutrophils indispensable escorts, or interchangeable partners within a broader platelet-dominated survival strategy?

Collectively, neutrophil–CTC partnerships illustrate how innate immune cells can be co-opted during metastasis, yet they also exemplify the field’s central limitation. Most evidence demonstrates strong association rather than definitive causation. Dissecting whether neutrophils initiate metastatic competence or selectively associate with already aggressive CTC subsets remains a critical unmet need (Ref. 61). By synchronizing immune evasion, vascular adhesion and microenvironmental remodeling, neutrophils significantly amplify metastatic efficiency, highlighting a critical axis for therapeutic intervention (see Table 2). Targeting this mechanism could disrupt CTC survival, extravasation and pre-metastatic niche formation simultaneously, revealing a high-value vulnerability in the metastatic cascade.

**Table 2.** Neutrophil-mediated mechanisms facilitating CTC survival and metastasis

Mechanism	Molecular mediators	Functional consequences	Conceptual insight/therapeutic implications
NET formation in TME and pre-metastatic niches	G-CSF, IL–8, HMGB1, platelet-derived CXCL4/CCL5; ROS; PAD4; neutrophil elastase; MPO	Chromatin extrusion forms DNA-histone-protease lattices; ECM remodeling exposes adhesion ligands; chemotactic recruitment of immune cells	NETs act as pre-metastatic architects, actively shaping tissues for CTC colonization. **Proposed:** PAD4 or NET scaffolding inhibitors
Platelet–neutrophil crosstalk amplifying NETosis	P-selectin–PSGL–1, CD40–CD40L, platelet-derived TGF-β, ADP, TXA2	Enhances NET formation and pro-thrombotic loops	Establishes a self-reinforcing thromboinflammatory circuit. **Proposed:** P-selectin or CD40L blockade
Tumor cell entrapment in NET scaffolds	Histones, neutrophil elastase, MPO, DNA backbone	Physical capture of CTCs; adhesion under shear stress; vascular docking	NETs serve as dynamic traps, prolonging CTC circulation and facilitating extravasation. **Proposed:** DNase or histone-neutralizing therapy
NET-driven thromboinflammation	Histones, tissue factor, thrombin, platelet activators	Microthrombosis around tumor cells; local immune shielding	Protects CTCs from NK/T cell clearance; creates survival niches. **Proposed:** anticoagulants (heparin, FXa inhibitors) in combination with NET disruption
Neutrophil hitchhiking	ICAM–1, β2 integrins, cytokines, ROS, proteases	Formation of neutrophil–CTC clusters; protection from shear stress, anoikis, and immune attack	Converts neutrophils into mobile metastatic escorts, enhancing CTC immune evasion. **Proposed:** adhesion molecule blockade
Pre-metastatic niche conditioning by neutrophils	NET-associated proteases, chemokines (CXCL1/2/5), ECM remodeling enzymes	Increased vascular permeability; fibroblast priming; recruitment of immunosuppressive cells	Neutrophils act as ‘niche engineers’, shaping tissue before CTC arrival. **Proposed:** early intervention to prevent metastatic organotropism
**Mechanism**	**Molecular Mediators**	**Functional Consequences for CTCs and TME**	**Conceptual depth/therapeutic implication**
**NET formation in TME and pre-metastatic niches**	G-CSF, IL–8, HMGB1, platelet-derived CXCL4/CCL5; ROS; PAD4; neutrophil elastase; MPO	Chromatin extrusion forms DNA-histone-protease lattices; ECM remodeling exposes adhesion ligands (integrins, selectins); creates chemotactic fields for monocytes, macrophages, platelets	NETs are not byproducts but **pre-metastatic architects**, reprogramming distal tissues into ‘fertile soil’ for CTC colonization. Targeting PAD4 or NET scaffolding proteins could disrupt niche priming.
**Platelet–neutrophil crosstalk amplifying NETosis**	Platelet P-selectin–neutrophil PSGL–1; CD40–CD40L; platelet-derived TGF-β, ADP, TXA2	Enhanced chromatin decondensation, oxidized DNA-rich NETs, pro-thrombotic and inflammatory loops	Establishes a **self-reinforcing thromboinflammatory circuit**, transforming innate immune defense into metastasis-permissive scaffolding. Blocking P-selectin or CD40L may interrupt this axis.
**Tumor cell entrapment in NET scaffolds**	Histones, neutrophil elastase, MPO, DNA backbone	Physical capture of CTCs, adhesion under shear stress, vascular docking	NETs act as **dynamic biomechanical traps** for CTCs, prolonging vascular dwell time and facilitating extravasation. DNase or histone-neutralizing strategies could dissolve these traps.
**NET-driven thromboinflammation**	Histones, tissue factor, thrombin, platelet activators	Localized platelet aggregation, fibrin deposition, microthrombosis surrounding tumor cells	Forms a **biochemical shield** against NK and T cell clearance; thrombi serve as pro-survival niches. Anticoagulant strategies (heparin, FXa inhibitors) may synergize with NET disruption.
**Neutrophil hitchhiking with CTCs**	Direct adhesion (ICAM–1, β2 integrins); cytokines; ROS; proteases	Formation of neutrophil–CTC clusters; protection from shear stress, anoikis, and immune surveillance	Converts neutrophils into **mobile metastatic escorts**, enhancing CTC immune evasion and vascular arrest. Blocking adhesion molecules or neutrophil–tumor clustering could dismantle this survival alliance.
**Pre-metastatic niche conditioning by neutrophils**	NET-associated proteases, chemokines (CXCL1, CXCL2, CXCL5), ECM remodeling enzymes	Increased endothelial permeability; stromal fibroblast priming; recruitment of immunosuppressive cells	Neutrophils act as **‘niche engineers’,** shaping tissue before CTC arrival. Early intervention at this stage may prevent metastatic organotropism.

## Consequences of platelet-leukocyte crosstalk in tumor progression

The interplay between platelets and leukocytes is increasingly recognized as a powerful regulator of tumor immune evasion, metastatic efficiency, and vascular remodeling (Ref. 13). However, this axis should not be viewed as uniformly pro-tumorigenic. Platelet–leukocyte aggregates exhibit significant phenotypic diversity, and their functional consequences vary with tumor type, disease stage and host immune context (Ref. 62). While extensive experimental evidence supports their role in immune suppression and pre-metastatic niche formation, clinical attempts to therapeutically disrupt these interactions have yielded inconsistent outcomes, highlighting the complexity of translating mechanistic insights into effective interventions (Refs 32, 45).

Rather than acting as singular drivers of metastasis, platelet–leukocyte interactions appear to function as amplifiers of existing malignant programs, reinforcing immune evasion, thromboinflammation and vascular dysfunction once systemic disease is established (Refs 13, 63). This distinction is critical, as it reframes these interactions from primary initiators to conditional enablers of metastatic progression.

### Immune modulation and suppression

Platelet-leukocyte aggregates act as highly dynamic, mobile immunoregulatory hubs, orchestrating a complex network of immune suppression within the TME and systemically (Ref. 13). Platelets actively ‘educate’ leukocytes through direct receptor-ligand interactions and soluble mediator release. Engagement of platelet P-selectin with leukocyte PSGL-1, CD40L-CD40 binding and integrin-mediated adhesion triggers intracellular signaling in neutrophils, monocytes and lymphocytes, reprogramming their phenotype toward immunosuppressive or pro-tumorigenic states (Ref. 64).

Neutrophils adopt an N2-like phenotype, producing anti-inflammatory cytokines, ROS and NETs that both suppress cytotoxic immune cells and remodel the extracellular environment to favor tumor persistence (Refs 65, 66, 67). Monocytes and macrophages polarize to the M2-like phenotype, characterized by VEGF, IL-10 and TGF-β secretion, promoting angiogenesis, matrix remodeling and immune evasion (Ref. 68). Platelet-derived TGF-β, thromboxane A2 and CXCL chemokines amplify these effects, inducing T-cell exhaustion, downregulating cytotoxic CD8^+^ T-cell function and expanding regulatory T-cell populations (Refs 62, 69).

The synergy of platelet and leukocyte crosstalk transforms the TME into an immune-privileged niche, where tumor cells are shielded from NK cell-mediated lysis, cytotoxic T-cell surveillance and antibody-dependent cellular cytotoxicity (Refs 13, 70). Importantly, this immune modulation is spatiotemporally orchestrated: platelets rapidly coat emerging tumor cells and CTCs, while recruited leukocytes sustain and amplify immunosuppressive signaling, creating a persistent protective network that supports tumor survival, growth and metastatic dissemination.

This dynamic axis not only facilitates local tumor immune escape but also establishes systemic immunomodulatory circuits, enabling pre-metastatic niche formation and enhancing the ability of disseminated tumor cells to colonize distant organs. Disrupting this platelet-leukocyte-mediated immunosuppressive network therefore represents a high-priority therapeutic target to reinstate anti-tumor immunity and reduce metastatic potential.

### Pre-metastatic niche formation

The platelet-leukocyte axis functions as a systemic architect of pre-metastatic niche formation, orchestrating molecular and cellular events that prime distant tissues for tumor colonization (Refs 13, 32, 45). Platelet-coated CTCs act as mobile signaling hubs, carrying pro-metastatic cues to remote sites (Ref. 71). Through the release of extracellular vesicles (EVs), chemokines (e.g., CXCL5, CCL2), and platelet-derived growth factors, these complexes condition endothelial cells, remodel the ECM and induce local stromal cells to adopt a pro-tumorigenic phenotype (Ref. 72).

This coordinated orchestration ensures that distant organs are pre-conditioned long before tumor cell arrival, creating a permissive ‘soil’ for metastatic seeding (Ref. 45). Importantly, the pre-metastatic niche is not a passive consequence of tumor dissemination, but an active, platelet-leukocyte-driven construct, integrating immune modulation, ECM remodeling, vascular adhesion and thromboinflammatory signaling. By establishing this systemic scaffold, platelets and leukocytes extend the temporal window for CTC survival, potentiate extravasation efficiency, and amplify metastatic potential – highlighting a critical therapeutic vulnerability for interventions aimed at disrupting metastatic niche formation.

### Thrombosis and vascular remodeling

The crosstalk between platelets and leukocytes establishes a pro-thrombotic, pro-inflammatory vascular milieu that is central to metastatic progression. Platelet aggregates, in concert with NETs and leukocyte-derived tissue factor, initiate microvascular thrombosis, which serves as both a physical scaffold and a signaling hub for CTCs (Ref. 50). These thromboinflammatory networks increase vascular permeability, exposing adhesion molecules (ICAM-1, VCAM-1, E-selectin) and extracellular matrix components that facilitate CTC arrest, adhesion and subsequent extravasation.

Simultaneously, the local endothelium undergoes activation and remodeling, characterized by upregulation of pro-coagulant and pro-inflammatory mediators, endothelial-to-mesenchymal transition, and enhanced expression of chemokines that recruit additional platelets and leukocytes (Ref. 56). This feed-forward loop amplifies vascular dysfunction, sustaining chronic inflammation while creating a permissive microenvironment for tumor colonization.

By converting the vasculature into a dynamic metastatic conduit, platelet-leukocyte interactions integrate thrombotic, inflammatory and adhesive cues, reinforcing the systemic network that supports tumor dissemination (Ref. 73). This vascular reprogramming represents a critical mechanistic link between immune evasion, pre-metastatic niche formation, and metastatic efficiency – highlighting an underexplored yet therapeutically targetable vulnerability in cancer progression.

### CTC survival and metastatic seeding

CTCs navigate a hostile circulatory environment, where shear stress, immune surveillanc, and anoikis threaten their survival (Refs 34, 74). The platelet-leukocyte axis orchestrates a multifaceted protective network that transforms this perilous journey into a highly efficient metastatic process (Refs 13, 75). Platelets are the first responders: they rapidly adhere to CTCs, forming a protective coating that masks tumor-associated antigens and shields them from NK cells and cytotoxic T lymphocytes (Refs 26, 76). Yet, this interaction extends well beyond a simple physical shield.

Sun et al. (Ref. 76) revealed that platelet–CTC adhesions initiate a profound transcriptional reprogramming of tumor cells, conferring active immune resistance rather than mere concealment. Mechanistically, direct platelet contact triggers the FAK/JNK/c-Jun signaling cascade, culminating in the upregulation of the inhibitory checkpoint ligand CD155 on CTCs. Functionally, CD155 engages TIGIT receptors on NK cells, silencing their cytotoxic machinery, while bypassing CD96 and DNAM1, two other potential CD155 partners. This selective engagement creates a unidirectional immunosuppressive axis that cripples innate immunosurveillance. Clinically, the presence of CTC–platelet aggregates correlates with shortened progression-free and overall survival in hepatocellular carcinoma, elevating them from a mechanistic curiosity to a prognostically meaningful biomarker. Moreover, therapeutic blockade of the CD155–TIGIT axis with neutralizing antibodies reinstates NK cell activity and markedly attenuates metastatic colonization in preclinical models.

Thus, platelets act not merely as passive coverings but as active architects of immune privilege, equipping CTCs with molecular tools to survive hematogenous transit and colonize distant sites. In parallel, neutrophils are recruited into these aggregates, ‘hitchhiking’ alongside CTCs to provide both mechanical buffering against shear forces and an additional layer of immune shielding, thereby reinforcing a cooperative metastatic niche. Together, these platelet–CTC–leukocyte alliances exemplify how hematologic partners are co-opted to convert a perilous circulatory journey into a fertile ground for metastatic seeding.

## Current therapeutic strategies targeting TME crosstalk

The platelet-leukocyte axis within the TME represents a central orchestrator of metastasis, immune evasion, thromboinflammation and pre-metastatic niche formation. Tumor cells exploit this dynamic network to evade immune surveillance, facilitate CTC survival and prime distant organs for colonization (Ref. 13). As such, this axis has emerged as a compelling therapeutic target. Current strategies largely aim to disrupt platelet activation, inhibit leukocyte recruitment or attenuate their bidirectional crosstalk, thereby interfering with the pro-tumorigenic and pro-thrombotic milieu that fuels metastatic progression.

### Conventional anti-platelet or anti-inflammatory approaches

Traditional interventions have focused primarily on broad pharmacological modulation of platelets and inflammatory pathways. Anti-platelet agents, such as aspirin or P2Y12 inhibitors, aim to inhibit platelet activation, reduce aggregation and attenuate interactions with CTCs (Refs 77, 78, 79). By interfering with platelet coating of tumor cells, these agents partially impair immune shielding, thromboinflammatory signaling, and platelet-driven recruitment of neutrophils and monocytes.

Anti-inflammatory approaches – including corticosteroids, NSAIDs and targeted cytokine inhibitors – aim to suppress leukocyte activation and recruitment, reducing NET formation and downstream immunosuppressive signaling (Refs 80, 81, 82). Such interventions can mitigate local tumor-promoting inflammation and blunt the immunosuppressive remodeling of the TME, although their systemic delivery often results in off-target effects and limited efficacy within pre-metastatic niches.

Complementary strategies target immune checkpoints and modulatory cytokines indirectly influenced by platelet-leukocyte crosstalk. For example, blockade of TGF-β or PD-L1 signaling can restore cytotoxic T-cell activity and counteract M2 macrophage polarization, partially reversing the immune suppression orchestrated by platelet-coated leukocytes (Refs 13, 83). These approaches highlight that modulation of even a single node within the platelet-leukocyte network can reduce metastatic potential and tumor progression in preclinical models.

However, despite strong mechanistic rationale, conventional antiplatelet therapies remain fundamentally limited: they act systemically rather than locally, often fail to temporally align with the dynamic platelet–leukocyte interactions, and cannot simultaneously target multiple synergistic pro-metastatic mechanisms (Ref. 13). Moreover, direct administration of antiplatelet agents without targeted delivery substantially increases the risk of hemorrhagic complications, a particularly critical concern in cancer patients who may already face thrombocytopenia or coagulopathy (Ref. 13). As a result, while CTCs continue to exploit platelet shielding, NET scaffolds and microthrombotic niches to survive circulation, evade immune detection and establish distant metastases, therapeutic strategies that go beyond untargeted systemic platelet inhibition are urgently needed.

This unmet need underscores the importance of next-generation therapeutics that are multi-modal, spatiotemporally precise and capable of selectively disrupting platelet-leukocyte crosstalk within the TME – strategies that could transform the prevention of metastasis and improve patient outcomes.

### Limitations of current therapies

Despite strong mechanistic rationale, conventional anti-platelet and anti-inflammatory strategies are fundamentally constrained by systemic delivery, incomplete pathway inhibition and temporal discordance with the dynamic metastatic cascade. Anti-platelet agents, including aspirin and P2Y12 inhibitors, effectively reduce thrombotic events but fail to fully abrogate platelet-mediated immune shielding, neutrophil recruitment and NET formation (Ref. 84). Moreover, sustained systemic platelet inhibition carries a high risk of hemorrhagic complications, limiting the intensity and duration of therapy that can be safely applied in cancer patients (Ref. 85).

Similarly, anti-inflammatory drugs – ranging from corticosteroids to cytokine inhibitors – lack spatiotemporal precision, often failing to reach nascent pre-metastatic niches or rapidly counteract acute platelet-leukocyte activation events (Refs 86, 87, 88). This temporal mismatch allows CTCs to exploit transient protective interactions, which collectively enhance survival, immune evasion and metastatic seeding.

Crucially, most current approaches are monolithic, targeting individual molecular pathways rather than the integrated, multi-nodal platelet-leukocyte network. This leaves significant pro-metastatic circuitry intact, including thromboinflammatory feedback loops, chemokine-driven leukocyte polarization and endothelial priming for extravasation. Consequently, tumor cells retain the capacity to exploit systemic and local immunomodulatory mechanisms, limiting the efficacy of conventional pharmacotherapy in halting metastatic progression.

These limitations underscore the pressing need for next-generation, tumor microenvironment-targeted interventions that are multi-modal, temporally adaptive and locally responsive, capable of selectively disrupting platelet-leukocyte crosstalk while preserving systemic hemostasis. Such strategies would not only inhibit immune evasion and metastatic niche formation but also create a precision anti-metastatic paradigm that aligns therapeutic action with the dynamic biology of tumor dissemination.

## Next-generation therapeutic concepts

Conventional anti-platelet and anti-inflammatory strategies are limited by systemic delivery, lack of temporal precision and incomplete inhibition of the multi-nodal platelet–leukocyte–tumor network. Recent evidence highlights the critical role of NETs in facilitating tumor immune evasion and metastasis (Ref. 89). Consequently, therapies that selectively target platelet-leukocyte interactions and NET formation in a spatiotemporally controlled manner are urgently needed.

Nanotechnology offers a versatile platform to achieve this goal. Functionalized nanoparticles can locally disrupt platelet-mediated immune shielding, neutrophil recruitment and NET scaffolds, while minimizing systemic exposure (Refs 90, 91, 92). Surface modification with ligands targeting adhesion molecules, such as P-selectin, PSGL-1 or CD40L, allows selective engagement with tumor-associated platelets and CTCs, creating opportunities for targeted payload delivery.

### Nanoparticles targeting platelet-leukocyte crosstalk

Nanoparticle-based systems offer a versatile platform to interrogate and therapeutically modulate the platelet–leukocyte–tumor axis, a central orchestrator of immune evasion, thromboinflammation and metastatic dissemination (Ref. 13). These systems can be engineered to deliver combinatorial payloads – including anti-platelet agents (e.g. P2Y12 inhibitors), immunomodulators (e.g. PD-1/PD-L1 antibodies), cytotoxic drugs and NET-degrading enzymes or iron-regulatory molecules – directly to primary tumors, CTCs, and pre-metastatic niches (Refs 89, 93). By simultaneously targeting these interconnected components, nanoparticles hold the potential to suppress tumor survival, disrupt platelet-mediated immune shielding and restore effective anti-tumor immunity.

Functionalization with ligands recognizing adhesion molecules such as P-selectin, PSGL-1, CD40 or CD40L enables precise engagement with activated platelets, leukocytes and CTCs. These interactions allow nanoparticles to locally perturb platelet–immune crosstalk, reducing NET formation, platelet-driven leukocyte recruitment and niche conditioning, while limiting systemic exposure and off-target effects (Refs 13, 94). Beyond adhesion blockade, nanoparticles may serve as precision delivery vehicles for anti-inflammatory agents, anti-thrombotics, ROS scavengers and other immunomodulatory payloads, enabling spatiotemporally controlled intervention aligned with the transient kinetics of metastatic progression.

Mechanistically, nanoparticles can conceptually disrupt the temporal program by which platelets coordinate CTC survival and immune evasion, attenuating both intravascular persistence and early metastatic colonization (Refs 24, 45). By sensing biochemical cues such as ROS, thrombin or inflammatory cytokines, multifunctional nanoparticles can dynamically adapt payload release to local TME conditions, offering a framework for rational, context-responsive anti-metastatic therapy.

It is important to emphasize that these strategies remain largely exploratory. While preclinical and model-based studies suggest profound potential, in vivo specificity, safety and reproducibility remain critical challenges. Nevertheless, this conceptual framework positions nanoparticles not merely as delivery vehicles but as experimental tools that integrate mechanistic insight with translational innovation, bridging platelet-leukocyte biology and next-generation therapeutic design.

### Hydrogels and localized TME modulation

Smart hydrogel systems have emerged as investigational biomaterial platforms for localized modulation of the tumor microenvironment (TME) in preclinical models (Refs 95, 96, 97). By incorporating responsiveness to stimuli such as ROS, enzymatic activity, pH or inflammatory cytokines, hydrogels can be engineered to release therapeutic agents in a spatially confined manner at sites of tumor–immune–platelet interaction (Refs 98, 99). Within a conceptual framework, these systems offer a means to explore how localized perturbation of inflammation, thrombosis or immune signaling may influence metastatic competence.

In addition to serving as delivery matrices, hydrogels are often described as bioactive scaffolds capable of shaping local tissue responses (Ref. 100). Experimental studies suggest that such materials can modulate oxidative stress, neutrophil activation and endothelial integrity, thereby influencing CTC adhesion and vascular trapping (Refs 101, 102). However, these observations are largely derived from controlled model systems, and their relevance to heterogeneous human tumors remains to be established.

The conceptual integration of hydrogels with nanoparticle-based approaches represents a hypothesis-driven, dual-level strategy, combining localized microenvironmental modulation with systemic targeting. While theoretically appealing, this multi-layered approach should be interpreted as a design paradigm rather than a validated therapeutic solution, given the substantial challenges associated with material biocompatibility, immune interactions and translational scalability.

## Conceptual and emerging strategies targeting the tumor microenvironment and metastatic cascade

This section presents a theoretical design framework for multi-stimuli-responsive nanoparticle systems, intended to illustrate how distinct tumor- and niche-associated biochemical cues could be exploited to improve spatial and temporal control of drug delivery. These strategies are conceptual in nature and are not intended to imply immediate clinical applicability.

### Design of multi-stimuli responsive nanoparticles

#### pH-sensitive drug release in the tumor microenvironment

The acidic extracellular pH characteristic of many solid tumors (approximately pH 6.5–6.8) arises from altered metabolism, hypoxia, and impaired perfusion (Refs 103, 104). In experimental nanomedicine research, this feature has been widely explored as a trigger for conditional drug release using pH-labile linkers or acid-sensitive polymers (Refs 105, 106, 107). Conceptually, such systems may enhance intratumoral drug localization while reducing systemic toxicity, a recurring limitation of conventional chemotherapy.

Importantly, pH-responsive behavior should be viewed as one component of a broader targeting logic, rather than a stand-alone solution. While preclinical studies demonstrate improved tumor accumulation and uptake (Refs 108, 109), heterogeneity in tumor acidity and off-target activation remain significant barriers. Accordingly, pH-sensitive designs primarily serve as proof-of-concept models informing the development of more selective delivery strategies.

#### Enzyme-sensitive release in pre-metastatic niches

Pre-metastatic niches are characterized by extracellular matrix remodeling, inflammatory signaling and enhanced protease activity, including elevated levels of matrix metalloproteinases (MMPs) released by activated neutrophils and amplified by NETosis (Refs 110, 111, 112). Enzyme-responsive nanoparticles incorporating MMP-cleavable linkers have therefore been proposed as context-aware delivery systems capable of releasing payloads selectively within these niches (Ref. 113).

Within a conceptual framework, enzyme-sensitive activation offers a means to localize therapeutic action to sites where platelet–leukocyte aggregates, ECM remodeling and immunosuppression converge. However, variability in protease expression and the risk of premature activation necessitate cautious interpretation of these approaches as exploratory design strategies, rather than established therapeutic modalities.

#### Targeted recognition of tumor vasculature

Functionalization of nanoparticles with ligands targeting P-selectin has been proposed as a strategy to enhance accumulation at activated endothelium and platelet-coated circulating tumor cells (Refs 114, 115). This design concept leverages inflammatory vascular signatures and the platelet ‘hitchhiking’ phenomenon to improve targeting specificity. In principle, such systems could enable simultaneous interaction with primary tumors and disseminating cells, but the robustness and safety of this approach in vivo remain incompletely understood.

### Multi-agent therapeutic payloads

To comprehensively disrupt tumor progression, metastasis and immune evasion, multi-stimuli-responsive nanoparticles can be loaded with complementary therapeutic agents that act synergistically within the TME, pre-metastatic niches and circulation. This multi-agent design enables simultaneous targeting of tumor cells, platelets and immune checkpoints, maximizing anti-metastatic efficacy while minimizing systemic toxicity.

#### Cytotoxic drugs for tumor lysis

pH-sensitive nanoparticles are designed to enable selective release of cytotoxic agents such as doxorubicin, paclitaxel or oxaliplatin within the acidic TME (pH ≈ 6.5–6.8). By concentrating chemotherapy directly at tumor sites, these nanoparticles enhance tumor cell apoptosis, reduce the shedding of CTCs and minimize off-target systemic effects. This localized cytotoxicity not only debulks the primary tumor but also interrupts the initial steps of metastasis by limiting the number of CTCs entering the bloodstream.

#### Anti-platelet agents to disrupt tumor-platelet crosstalk

Enzyme-sensitive release in pre-metastatic niches delivers anti-platelet drugs (e.g., P2Y12 inhibitors, clopidogrel derivatives) to inhibit platelet activation, aggregation and the platelet–CTC aggregate formation. By targeting platelets in both the tumor microenvironment and circulating pre-metastatic niches, this approach reduces neutrophil hitchhiking, NET-mediated CTC entrapment and thromboinflammatory remodeling, may contribute to disruption of a key mechanism of tumor immune evasion.

#### Immunomodulatory agents

Checkpoint inhibitors such as anti-PD-1 or anti-PD-L1 antibodies can be incorporated into the same nanoparticle system to locally restore T-cell cytotoxicity. Targeted delivery ensures that immune modulation occurs preferentially within the TME and pre-metastatic niches, enhancing anti-tumor responses without provoking systemic immune-related adverse effects. By combining immune activation with platelet disruption and cytotoxic therapy, the nanoparticles create a synergistic tri-modal attack on both primary tumors and metastatic seeds.

### Integrated multi-modal mechanism of action

The optimized nanoparticle strategy leverages spatiotemporal precision and multi-level targeting to disrupt the tumor-platelet-leukocyte-metastasis axis. pH-sensitive release ensures that cytotoxic agents such as doxorubicin or paclitaxel are preferentially delivered within the acidic tumor microenvironment, directly lysing tumor cells and reducing CTC shedding. Enzyme-sensitive responsiveness enables selective payload release in pre-metastatic niches, where matrix MMPs secreted by activated neutrophils and NETosis remodel the extracellular matrix. This targeted release disrupts platelet-leukocyte activation, prevents NET-mediated CTC entrapment and impedes metastatic niche formation.

Anti-P-selectin functionalization provides vascular and CTC specificity, exploiting the platelet ‘hitchhiking’ mechanism to guide nanoparticles to circulating tumor cells while also concentrating therapy at activated endothelium. The tri-modal payload – comprising cytotoxic drugs, anti-platelet agents and immune checkpoint inhibitors – simultaneously suppresses tumor survival, abrogates platelet-mediated immune shielding, and reactivates cytotoxic T-cell responses within both primary and secondary sites.

By integrating tumor-specific, niche-specific and circulation-specific targeting, this multi-modal system aims to concurrently modulate tumor cells, circulating tumor cells and the platelet–leukocyte–tumor axis, thereby disrupting multiple interconnected steps of metastatic progression. It transcends single-pathway therapies by synchronously addressing the cellular, vascular and immunological components of tumor progression, offering a paradigm-shifting approach for preventing metastatic dissemination and improving patient outcomes.

### Translational and clinical considerations

The translation of multi-stimuli-responsive, multi-agent nanoparticles into clinical practice necessitates careful optimization across multiple parameters. Nanoparticle physicochemical properties – including size, surface charge, hydrophobicity and circulation half-life – must be fine-tuned to maximize tumor and pre-metastatic niche accumulation, enhance platelet ‘hitchhiking’ for CTC targeting, and minimize renal clearance or off-target organ deposition. Surface functionalization with anti-P-selectin probes further improves specificity while reducing systemic exposure.

Safety profiling is critical, particularly given the inclusion of anti-platelet agents, cytotoxic drugs and immune checkpoint inhibitors. Strategies must mitigate risks of bleeding, thrombocytopenia, or immune overactivation, while ensuring that local therapeutic concentrations are sufficient to disrupt platelet-leukocyte crosstalk, NET formation and pre-metastatic niche establishment. Preclinical models should include comprehensive hematologic, immunologic and cardiovascular assessments, alongside pharmacokinetics and biodistribution studies.

Integration with current standard-of-care therapies – including chemotherapy, targeted therapy and immunotherapy – offers the potential for synergistic effects. The nanoparticle system could act as a precision adjuvant, reducing metastatic seeding and enhancing anti-tumor immunity while limiting systemic toxicity. Finally, scalable manufacturing under Good Manufacturing Practice (GMP) standards, reproducibility and regulatory compliance will be essential to move these sophisticated multi-modal platforms from the bench to clinical trials.

**Figure 2.**
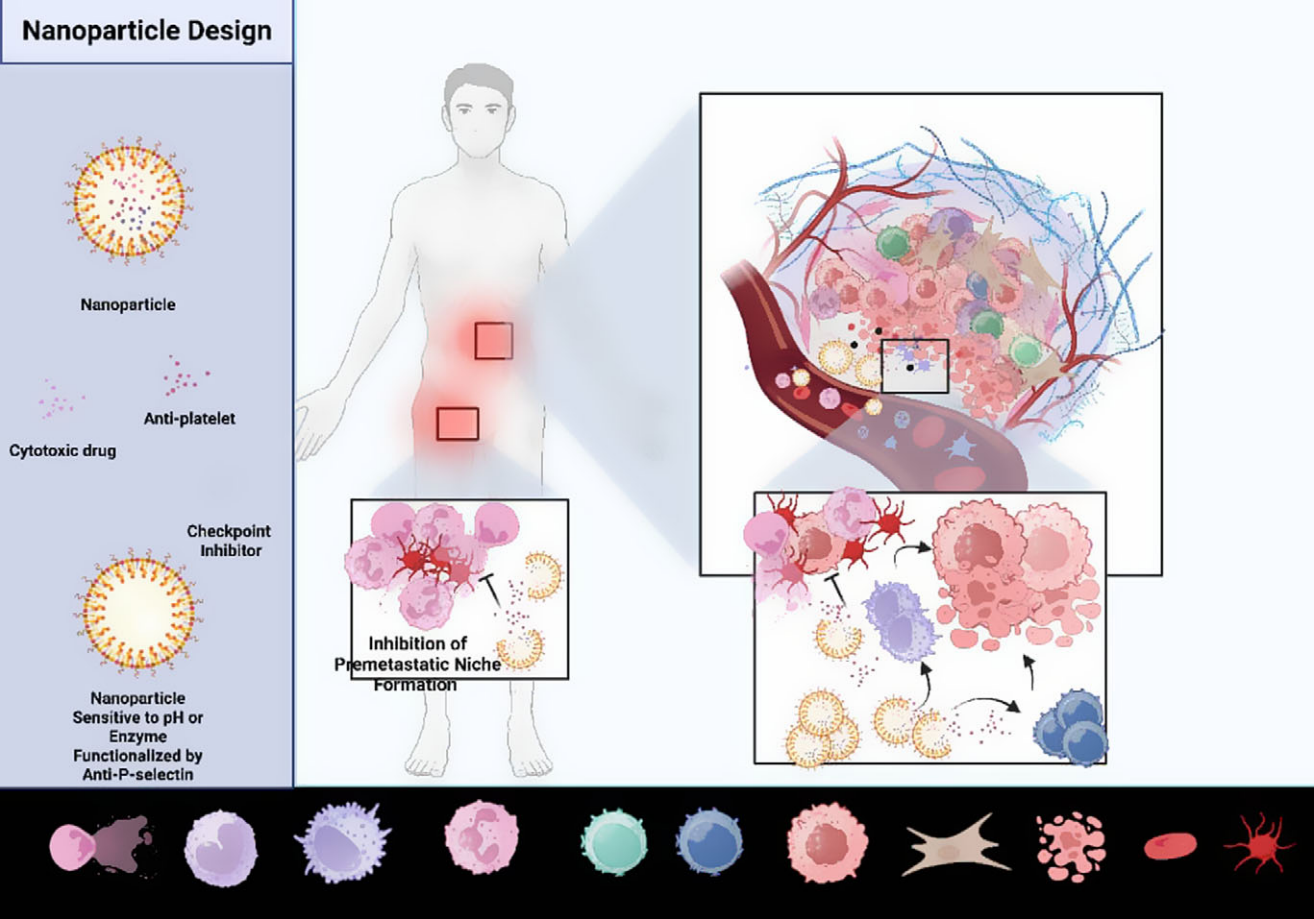
Design and mechanistic action of multifunctional tumor-targeted nanoparticles. *Note*: Left panel: Schematic of nanoparticles engineered to respond to enzymatic or acidic triggers in the tumor microenvironment. Functionalization with P-selectin enables selective targeting of tumor vasculature or platelet-bound CTCs. Nanoparticles carry cytotoxic agents (e.g., doxorubicin), antiplatelet drugs and immune checkpoint modulators. Right panel: Upon encountering tumor-specific triggers, nanoparticles release their payload locally. Proposed mechanisms include tumor cell killing, suppression of platelet activation and disruption of platelet–tumor and platelet–neutrophil crosstalk, reducing immune evasion and pre-metastatic niche formation. In addition, MMP-sensitive nanoparticles respond to NET-derived enzymes to prevent ECM remodeling and metastatic seeding. Figure 2 integrates established tumor biology with mechanistic therapeutic strategies, illustrating points of vulnerability in metastatic progression.

This framework positions multi-stimuli-responsive nanoparticles not merely as experimental tools, but as conceptual frameworks with potential translational relevance, capable of simultaneously targeting tumor cells, circulating tumor cells and pre-metastatic niches to prevent metastasis and improve patient outcomes (see Figure 2).

## Conclusion

### Platelet-leukocyte crosstalk as a central node in metastasis

The intricate interplay between platelets and leukocytes constitutes a central regulatory hub driving metastatic progression, immune evasion and systemic dissemination of tumor cells. Beyond their classical hemostatic and immune roles, these cellular networks are co-opted by tumors to form a dynamic, adaptive and multi-level pro-metastatic ecosystem. Platelet-coated CTCs, providing both physical protection against shear stress and immune evasion from NK cells and cytotoxic T lymphocytes. Concurrently, platelet-derived factors such as TGF-β, P-selectin and CD40L modulate leukocyte phenotype, induce NET formation, polarize macrophages toward M2-like pro-tumor states, and suppress T-cell cytotoxicity. This coordinated crosstalk establishes mobile, systemic immunosuppressive and thromboinflammatory networks, priming pre-metastatic niches through extracellular matrix remodeling, endothelial activation, and localized cytokine gradients. Far from being a passive byproduct, metastasis emerges as a highly orchestrated, multi-cellular program, wherein platelet-leukocyte cooperation serves as a critical fulcrum for both local tumor progression and distant organ colonization. Mapping this axis illuminates previously underappreciated vulnerabilities within the metastatic cascade, offering precise points of therapeutic intervention.

### Potential of nanoparticle-based, TME-targeted therapeutics

Next-generation nanoparticle platforms provide a precision-guided means to disrupt these multi-dimensional pro-metastatic circuits. By leveraging multi-stimuli responsiveness – pH sensitivity to the acidic tumor microenvironment and enzyme-triggered release in pre-metastatic niches enriched with MMPs – nanoparticles achieve spatiotemporally controlled delivery of cytotoxic, anti-platelet and immunomodulatory payloads. Functionalization with anti-P-selectin probes enhances selective targeting of tumor vasculature and activated platelets, while simultaneously enabling the platelet ‘hitchhiking’ mechanism to reach CTCs in circulation. These systems are capable of simultaneously dismantling platelet-leukocyte shields, neutralizing NET-mediated scaffolds, modulating leukocyte phenotype and restoring anti-tumor immune function. Importantly, this multi-modal targeting transforms therapeutic interventions from blunt, single-pathway approaches into adaptive, network-oriented strategies, capable of intercepting metastatic processes at multiple stages: from primary tumor invasion, to CTC survival in circulation, to pre-metastatic niche establishment in distant organs.

### Toward precision anti-metastatic therapy

The convergence of mechanistic insight, nanotechnology and smart biomaterial design lays the groundwork for truly precision anti-metastatic therapies. By integrating cytotoxic, anti-platelet and immunomodulatory actions within a single, tunable platform, it becomes possible to simultaneously target the tumor, the circulatory transit of CTCs and the pre-metastatic niche, thereby minimizing metastatic seeding while enhancing immune surveillance. Critical translational considerations include optimization of nanoparticle pharmacokinetics, surface properties, biocompatibility and dosing regimens to maximize efficacy while minimizing systemic toxicity and off-target platelet inhibition. Moreover, integration with existing therapeutic regimens – including conventional chemotherapy, immunotherapy and anti-thrombotic agents – offers opportunities for synergistic, multi-pronged intervention. Ultimately, by reconceptualizing metastasis as a networked, highly coordinated process, these approaches shift the paradigm from reactive, post-metastatic treatment to proactive, multi-nodal interception, opening a path toward clinically actionable strategies that may finally prevent metastatic disease rather than merely treat it.

## Data Availability

This is a review study, and it is not an original study.
